# Imiquimod Treatment Causes Systemic Disease in Mice Resembling Generalized Pustular Psoriasis in an IL-1 and IL-36 Dependent Manner

**DOI:** 10.1155/2016/6756138

**Published:** 2016-12-12

**Authors:** Pilar Alvarez, Liselotte E. Jensen

**Affiliations:** Department of Microbiology and Immunology, Lewis Katz School of Medicine at Temple University, 1158 MERB, 3500 N. Broad Street, Philadelphia, PA 19140, USA

## Abstract

Generalized pustular psoriasis (GPP) is a severe form of psoriasis that can be caused by missense mutations in the interleukin-36 (IL-36) receptor antagonist. In addition to neutrophil rich skin inflammation, GPP patients typically also experience anorexia, fever, malaise, and pain. The imiquimod-induced skin inflammation mouse model has rapidly become a popular way to study plaque psoriasis, which typically does not involve symptoms of systemic disease. In this model, neutrophil recruitment to the skin is dependent upon the inflammatory mediators IL-1, via its receptor IL-1R1, and IL-36*α*. Unexpectedly, we observed that mice also exhibited signs of anorexia (weight loss and decreased food intake), general malaise (decreased activity and loss of interest in building nests), and pain (nose bulging and hunched posture). A scoring system allowing quantitative comparisons of test groups was developed. Female mice were found to develop more severe disease than male mice. Furthermore, mice deficient in both IL-1R1 and IL-36*α* are nearly disease-free, while mice lacking only one of these inflammatory mediators have less severe disease than wild type mice. Hence, the imiquimod-induced skin inflammation mouse model recapitulates not only plaque psoriasis, but also the more severe symptoms, that is, anorexia, malaise, and pain, seen in GPP.

## 1. Introduction

Psoriasis represents a spectrum of inflammatory skin conditions ranging from mild to potentially life-threatening. Plaque psoriasis is the most common form and causes localized red scaly skin plaques. Generalized pustular psoriasis (GPP) is rare but is the most severe form of psoriasis and involves the formation of large sterile neutrophil pustules in the epidermis. The affected skin areas can cover the entire body and patients also suffer from anorexia, fever, malaise, and pain. Active disease flairs often appear abruptly and require hospitalization as the disease can be fatal. Progress towards developing a cure or satisfactory treatment approaches for GPP have been hampered by poor knowledge of disease mechanisms and the lack of experimental models in which drugs can be tested.

A major breakthrough in our understanding of GPP pathogenesis was made in 2011, when missense mutations in the interleukin-36 (IL-36) receptor antagonist, IL-36Ra, were identified in GPP patients [1, 2]. Several additional mutations have subsequently been identified (see [3] and the references therein). IL-36Ra is a classical receptor antagonist that acts as a natural inhibitor of the three related cytokines, IL-36*α*, IL-36*β*, and IL-36*γ*, by competitively binding to the common receptor IL-36R (see [1] and the references therein). The three IL-36 cytokines and IL-36R are related to the pleiotropic IL-1 cytokines, IL-1*α* and IL-1*β*, and their receptor, IL-1R1, respectively [4], and the IL-36 and IL-1 cytokines activate the same intracellular signaling mechanisms leading to, for example, production of cytokines such as CXCL1, CXCL2, and IL-8 by keratinocytes [1, 5–8]. This in turn promotes recruitment of neutrophils into the epidermis. Several GPP case studies, including some with IL-36Ra missense mutations, have reported successful treatment of both skin and systemic disease with granulocyte and monocyte adsorption apheresis, suggesting an important role for neutrophils in the disease (see [9, 10] and the references therein). Interestingly, some case studies have reported the efficacy of using IL-1 inhibitors, for example, the IL-1Ra or neutralizing antibodies, in GPP patients [11–14]. This suggests a role of both IL-36 and IL-1 in GPP pathogenesis.

The imiquimod-induced skin inflammation mouse model has been extensively used as a model of plaque psoriasis (reviewed in [15]). We have previously shown that IL-1 and IL-36 cooperate to promote neutrophil recruitment to the epidermis in this model [6, 7]. Here, we report the utilization of the imiquimod model to evaluate GPP related systemic disease such as anorexia, malaise, and pain. We also demonstrate dependence of these phenotypes upon IL-1 and IL-36, cytokines linked to GPP disease; hence, the model may be useful for mechanistic studies of GPP pathogenesis and drug development.

## 2. Materials and Methods

### 2.1. Mice

C57BL/6J and IL-1R1^−/−^ mice were obtained from Jackson Laboratory. The IL-36*α* ^−/−^ and IL-36*α* ^−/−^/IL-1R1^−/−^ mice have previously been described by us [6]. Wild type (C57BL/6J) and IL-1R1^−/−^ mice for experiments involving IL-36*α* ^−/−^ and IL-36*α* ^−/−^/IL-1R1^−/−^ mice were bred in-house. All procedures were approved by the Temple University Institutional Animal Care and Use Committee.

### 2.2. Imiquimod Treatment

Mice aged 7–10 weeks were anesthetized using isoflurane and denuded by trimming and Nair treatment as previously described [6, 7]. Fur and Nair were gently and thoroughly removed using water and WypAll wipes (Kimberly-Clark Professionals) before returning the mice to their cages. Daily imiquimod treatments were started the following day. Mice were treated daily with 62.5 mg (or as indicated) 5% imiquimod cream on a 6 cm^2^ area on the lower back for a total of four applications. Aldara (imiquimod) cream was obtained from Valeant (NJ, produced by 3M Health Care Limited, UK). Generic imiquimod cream was from Perrigo (Israel). Mice were matched for age and sex in each individual experiment. Following fur removal, control mice were sham-treated with Vaseline cream (Riedel-de Haen) or left untreated.

### 2.3. Disease Evaluations

Mice were weighed daily. Body temperature was measured using noninvasive infrared thermometers, for example, the Forehead and Ear Thermometer (Innovo). To facilitate evaluation of nest building and food intake, mice were housed individually starting at day 0 (e.g., day of the first imiquimod application) throughout the remainder of the experiments. This short period of isolation did not significantly affect behavior in untreated mice. Mice were fed DietGel 76A (ClearH_2_O) during the experiments and the entire plastic container with food was weighed daily (replaced every two days). Mice were allowed access to water* ad libitum*. Nestlet (Ancare) material was replaced every day, for example, at the time of imiquimod treatment. Facial expressions of pain were evaluated as described by others [16]. For total disease scores, changes in weight, food intake, and nest building were converted to scores. Each experiment, each including wild type controls, was conducted by the same person throughout the duration of the experiment to ensure consistent scoring. Mice in significant distress were euthanized.

### 2.4. Statistical Analyses

Each experiment contained four mice per test group and all experiments were performed at least 3 independent times. Statistical analyses were performed using paired and unpaired *t*-tests as appropriate. Differences in survival were evaluated using Mantel-Cox and Gehan-Breslow-Wilcoxon tests.

## 3. Results

### 3.1. Generic Imiquimod Cream Causes More Severe Disease Than Aldara Cream

**Figure 1:**
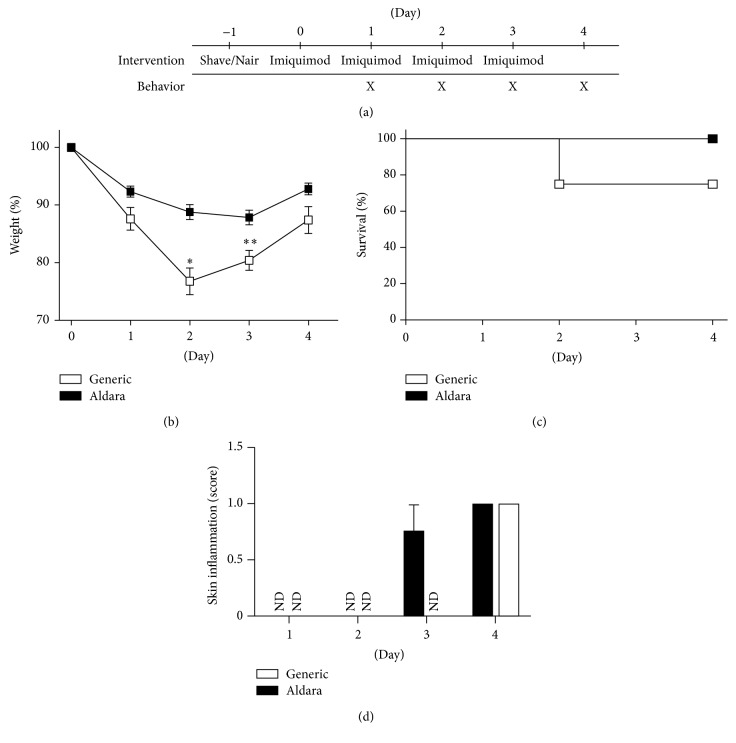
Generic imiquimod cream causes more severe disease than Aldara®. Male C57BL/6J mice (*n* = 4 per group) received daily treatments (a) with Aldara (black symbols) or generic (Perrigo®, open symbols) 5% imiquimod cream (62.5 mg). Weight ((b), expressed as percentage of values at day 0), survival (c), and skin inflammation (d) were monitored. Data from one representative experiment of at least 3 independent experiments is shown as means ± SD. ^*∗*^ *p* < 0.05; ^*∗∗*^ *p* < 0.01 (comparing Generic to Aldara at individual timepoints).

Previously, using the imiquimod-induced skin inflammation mouse model, we demonstrated that IL-1 and IL-36*α* cooperate to promote epidermal neutrophil recruitment [6]. Aiming to extend upon these studies, we serendipitously found that a generic version of imiquimod cream (Perrigo) caused significantly more severe phenotypes in terms of weight loss and survival than the brand name Aldara imiquimod cream (Figure 1).

### 3.2. Systemic Disease Can Be Scored for Anorexia, General Malaise, and Pain

**Figure 2:**
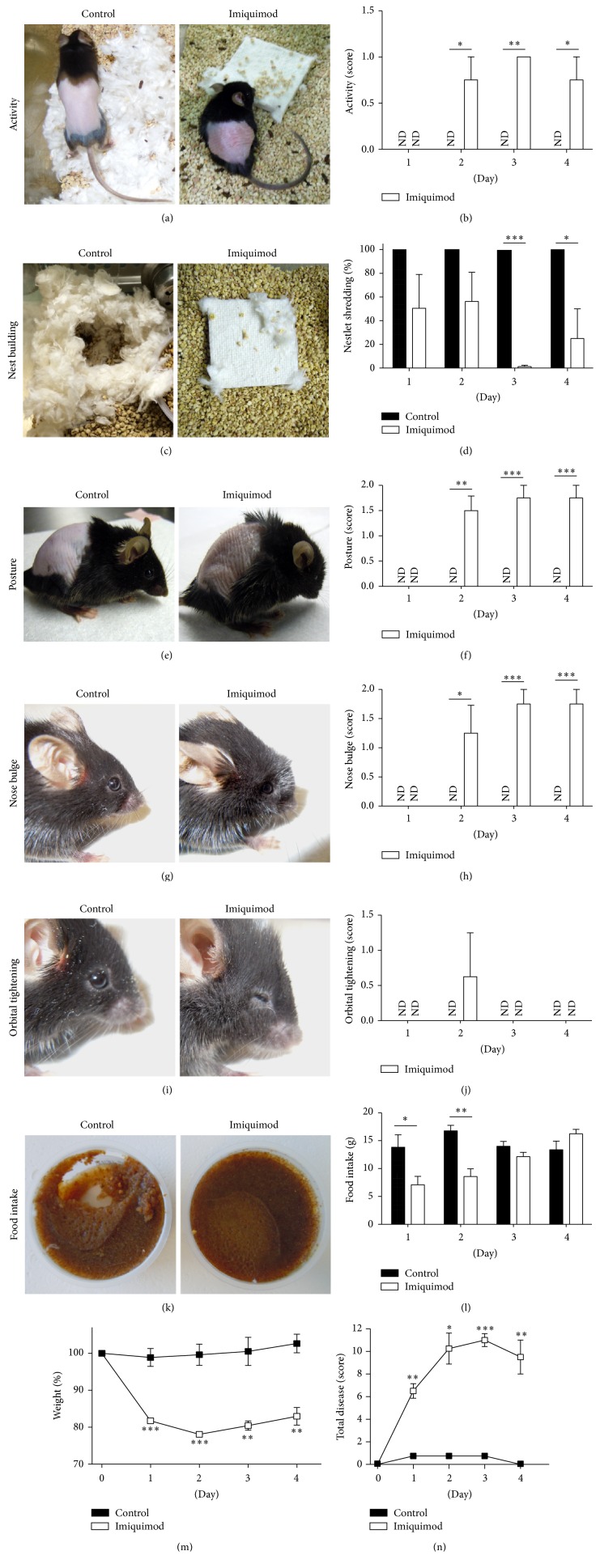
Topical imiquimod cream application causes systemic disease in mice resembling that observed in GPP patients. Male C57BL/6J mice (*n* = 4 per group) were treated daily with 62.5 mg 5% imiquimod cream (Perrigo, open bars and symbols) or were sham-treated (black bars and symbols). Behavioral changes (physical activity (a and b) and nest building (c and d)), pain (posture (e and f), nose bulging (g and h), and orbital tightening (i and j)), and anorexia (food intake (k and l) and mouse body weight (m)) were evaluated and scored as described in Table 1. Total disease scores (n) were calculated as the sum of individual scores for each mouse and group means (±SD) shown. ND: no disease detected, score = 0. Graphed data from one representative experiment of more than 3 independent experiments is shown as means ± SD. ^*∗*^ *p* < 0.05; ^*∗∗*^ *p* < 0.01; ^*∗∗∗*^ *p* < 0.001 (comparing imiquimod to control at individual timepoints). Pictures from independent experiments were taken at day 4.

During our earlier studies, we frequently observed behavioral changes, for example, reduced interest in nest building, in especially our wild type mice suggesting that the imiquimod treatments had effects beyond induction of skin inflammation. Infections and injuries can be associated with systemic responses such as anorexia, fever, general malaise, and muscle and joint pain as part of the body's efforts to reestablish homeostasis [17]. These physiological responses resemble some of the clinical manifestations reported in GPP patients; hence, to better characterize our behavioral observations and develop an approach allowing quantification of systemic disease responses, we established a scoring system to measure clinical manifestations often observed in GPP, that is, anorexia, general malaise, and pain (Figure 2 and Table 1). Malaise was evaluated by observing the physical activity of the mouse itself (Figures 2(a) and 2(b) and Table 1) and its progress in building a nest overnight (Figures 2(c) and 2(d) and Table 1). Pain was detected through posture (Figures 2(e) and 2(f) and Table 1) and facial expressions such as nose bulging (Figures 2(g) and 2(h) and Table 1) and orbital tightening (Figures 2(i) and 2(j) and Table 1). The use of facial expressions is based on a recent report correlating this to pain [16]. We found the nose bulge to be a common and easy-to-score outcome (Figures 2(g) and 2(h)). Orbital tightening scoring as 2 (Figure 2(i), imiquimod) was only observed in very sick animals and was an indicator of euthanasia being required. Due to weight loss, the swim test was not applied as a measure of depression/malaise. Similarly, as the back skin can become stiff in response to the imiquimod cream, the tail-suspension test was not used. To evaluate anorexia, we determined food intake (Figures 2(k) and 2(l) and Table 1) and body weight (Figure 2(m) and Table 1). Progressive changes in these disease parameters were observed in response to repeated imiquimod treatments over a 4-day period (Figure 2). Sums of individual disease parameters reflected overall disease progression (Figure 2(n)). Based on a single controlled experiment involving two groups treated, respectively, in the morning and the late afternoon, timing of the imiquimod cream applications did not affect disease outcomes (data not shown). Furthermore, we did not detect differences in temperature or between control mice that were either sham-treated with Vaseline cream or left untreated (data not shown).

### 3.3. Female Mice Develop More Severe Disease Than Male Mice

**Figure 3:**
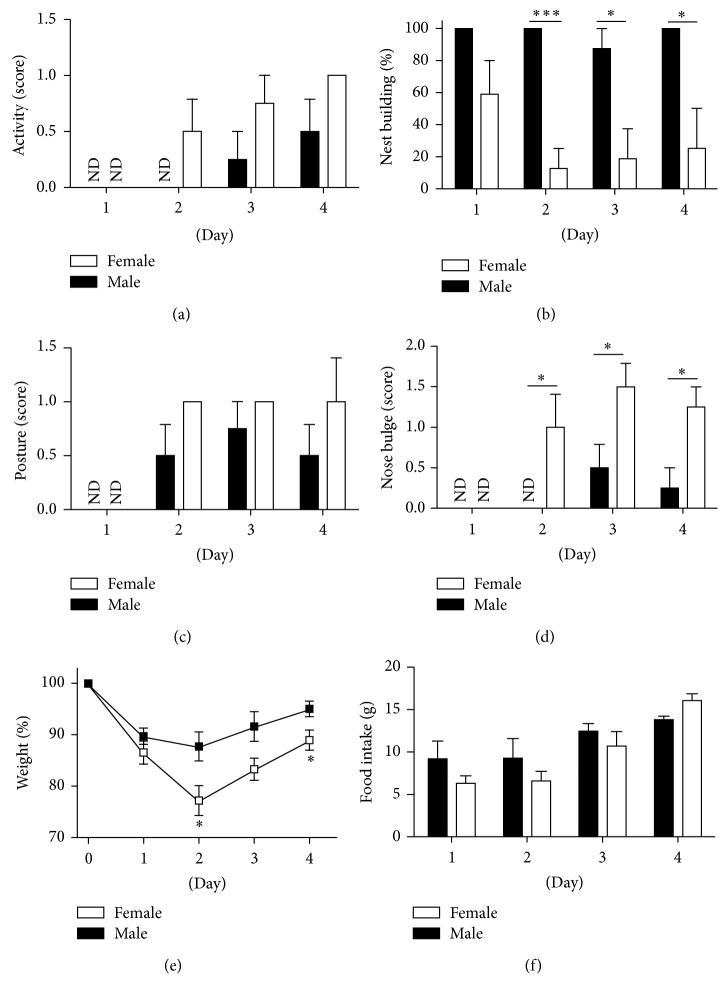
Female mice develop more severe disease than male mice in response to generic imiquimod cream. Wild type male (black bars and symbols) and female (open bars and symbols) mice (*n* = 4 per group) were treated with 31.25 mg imiquimod cream (Perrigo) once daily for a total of 4 applications. Activity (a), nest building (b), posture (c), nose bulging (d), weight (e), and food intake (f) were evaluated as described in Figure 2 and Table 1. ND: no disease detected, score = 0. Data shown (means ± SD) is from one representative experiment of at least 3 independent experiments. ^*∗*^ *p* < 0.05; ^*∗∗∗*^ *p* < 0.001 (comparing female to male at individual timepoints).

In some initial experiments using generic imiquimod cream and female mice, mice either died unexpectedly or had to be euthanized before the end of the experiments due to significant distress and dramatic weight loss (data not shown and data below). To compare male and female mice, we used a reduced dose of generic imiquimod cream. As expected based on the initial experiments, female mice exhibited several signs of more severe systemic disease than male mice (Figure 3). While the readouts for activity (malaise) and posture (pain) only revealed trends towards gender specific differences, nest building (malaise), weight (anorexia), and nose bulging (pain) were significantly more affected in female than in male mice (Figure 3). To the best of our knowledge, this is the first time sex specific differences in the imiquimod model are reported; however, such gender specific differences are in general agreement with the long established stronger immune responses observed in females [18].

It should be noted that we here used a fixed dose and not a treatment adjusted to the size of the individual mouse. This approach is in agreement with that employed when skin inflammation induced by imiquimod cream is examined (see [15] for references). Since female mice are smaller than male mice, the females were treated with a greater dose per gram body weight. Hence, it cannot be concluded whether female mice* per se* are more sensitive to the drug than male mice.

### 3.4. Systemic Disease Is IL-1R1 and IL-36*α* Dependent

**Figure 4:**
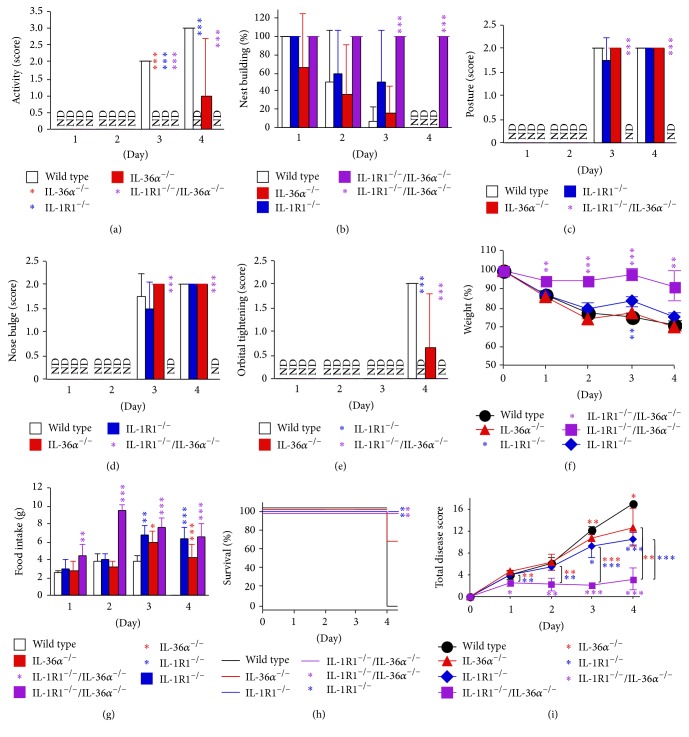
Systemic disease caused by imiquimod cream is IL-36*α* and IL-1R1 signaling dependent. Wild type (open bars, black circles, and lines), IL-36*α* ^−/−^ (red bars, triangles, lines, and statistics asterisks), IL-1R1^−/−^(blue bars, diamonds, lines, and statistics asterisks), and IL-36*α* ^−/−^/IL-1R1^−/−^ (purple bars, squares, lines, and statistics asterisks) female mice (*n* = 3–5 per group) were treated daily with 5% imiquimod cream (Perrigo) for 4 days. Activity (a), nest building (b), posture (c), nose bulging (d), orbital tightening (e), weight (f), food intake (g), and survival (h) were evaluated and scored as described in Table 1 and illustrated in Figure 2. ND: no disease detected, score = 0. Total disease scores (i) were calculated as the sum of individual scores for each mouse and group means (±SD) shown. ^*∗*^ *p* < 0.05; ^*∗∗*^ *p* < 0.01; ^*∗∗∗*^ *p* < 0.001 (compared to control at individual timepoints unless indicated otherwise with black bars). Data is shown from one representative experiment of 3 independent experiments.

We previously found that both IL-1R1 and IL-36*α* are important for the development of psoriasis-like skin inflammation in the imiquimod model [6, 7]; thus, we next examined their role in systemic disease (Figure 4). No differences between sham treated wild type, IL-1R1 knockout, IL-36*α* knockout, and IL-1R1/IL-36*α* double knockout mice were detected (data not shown). Mice lacking IL-1R1 (Figure 4, blue bars, lines, and symbols) exhibited significantly less overall disease due to diminished signs of malaise, pain, and anorexia compared to wild type mice (Figure 4, open bars and black lines and symbols) when treated with imiquimod. This is in agreement with the known role of IL-1*β* in inducing fever and pain [19, 20]. Interestingly, mice deficient in IL-36*α* also experienced milder disease (Figure 4, red bars, lines, and symbols). This effect was most pronounced in the IL-1R1/IL-36*α* double knockout mice, which exhibited largely normal behavior and no signs of pain (Figure 4, purple bars, lines, and symbols). Hence, the systemic disease induced by imiquimod in mice is dependent upon inflammatory mediators known to play a role in human GPP.

## 4. Discussion

The imiquimod-induced skin inflammation mouse model is frequently used to study mechanisms of psoriasis pathogenesis and for testing new drugs. GPP is a severe form of psoriasis, which is also associated with anorexia, fever, malaise, and pain. Here, using the imiquimod model, we demonstrate that mice develop symptoms of systemic disease (Figures 1 and 2) resembling those observed in GPP, that is, anorexia, malaise, and pain. Furthermore, we have developed a scoring system (Table 1) that allows quantitative comparisons between test groups (Figures 3 and 4). The IL-1/IL-36 dependence of the systemic phenotypes in this mouse model (Figure 4) suggests strong correlation to human GPP pathogenesis, as IL-36Ra missense mutations are now known to cause GPP (see [1–3] and the references therein) and at least some GPP patients experience dramatic improvement of their disease when treated with IL-1 inhibitors [11–14].

While the observed phenotypes are largely IL-1 and IL-36*α* dependent (Figure 4), mild anorexia and weight loss were still observed in IL-1R1/IL-36*α* double knockout mice (Figures 4(f) and 4(g)). This suggests additional mechanisms contributing to these disease outcomes. Interestingly, onset of anorexia and weight loss is very rapid, that is, apparent already on day 1 (Figures 2(k)–2(m)), and disease severity improves during the experiment (days 3-4, Figures 2(k)–2(m)). Hence, the IL-1R1/IL-36*α* independent effect upon food intake and body weight appears to involve a transient induction/response and/or negative regulator. The identification of this mechanism(s) will require further examination.

It has been reported that isostearic acid in Aldara contributes to the inflammatory response induced by the cream [21]. Interestingly, the generic cream produced by Perrigo does not contain isostearic acid; hence, this compound cannot explain why the generic form of the drug causes more severe systemic disease than the brand name cream (Figure 1). However, the Perrigo cream contains oleic acid, which is a common and naturally occurring fatty acid in, for example, adipose tissue and olive oil. While oleic acid does not itself promote inflammation, it has been reported that it can enhance (2–4-fold) IL-23 production by dendritic cells in response to the inflammatory mediator LPS [22]. The role of the IL-23/IL-17 axis and cross-communication between dendritic and epithelial cells in the imiquimod model in an IL-36 receptor dependent manner has been reported [23, 24]. Whether the Perrigo cream contains sufficient oleic acid to potentiate the* in vivo* inflammatory response of dendritic cells to imiquimod in the model remains to be determined.

While oleic acid in generic imiquimod cream may explain why this has higher activity than Aldara (Figure 1), imiquimod appears to be the primary driver of the systemic phenotypes as these can be observed with Aldara, generic imiquimod cream, and soluble imiquimod. While this manuscript was in preparation, an independent study reported that imiquimod (Aldara or soluble imiquimod) applied specifically to the skin decreased burrowing activity [25], which involves searching for hidden food. This outcome may be a consequence of malaise (activity and nest building) and anorexia (food intake) as reported here, for example, Figure 2. The pain experienced by the mice may be an additional contributing factor. Since burrowing is no longer a human activity, the here used readouts (Table 1 and Figure 2) may be more useful, when imiquimod is used to model human disease outcomes, including anorexia, malaise, and pain as seen in GPP patients.

The reported decreased burrowing activity in response to topical imiquimod was associated with gene expression changes and inflammation in the brain [25]. The IL-36 system of cytokines and receptor is expressed in the brain [26–29]; however, early studies seeking to identify IL-36 functions failed at detecting effects of recombinant proteins both* in vivo *and* in vitro* [26, 27]. In light of the recent discovery that IL-36 activity is enhanced by removal on N-terminal amino acids from the recombinant IL-36 proteins [30], it is possible that the shorter IL-36 cytokines could drive neuroinflammation [28]. Lack of processing of IL-36*γ* in experimental autoimmune encephalomyelitis has been used to explain why IL-36*γ* and IL-36R KO mice develop the same degree of disease as wild type mice, despite high levels of the cytokine being expressed in the brain during disease [28]. Given the observed effect of IL-36*α* deletion in the outcome of imiquimod treatment (Figure 4), it is possible that in this model system IL-36*α* acts directly on the brain. Further studies will be required to determine exactly how IL-36*α* promotes systemic disease.

In summary, treatment of mice with imiquimod cream causes systemic phenotypes resembling those observed during infections and in patients with GPP. The imiquimod-induced skin inflammation mouse model with behavioral observations, as described here, may represent a suitable model in which novel therapies can be developed to manage GPP. Since IL-1 and IL-36 are known to be involved in the human GPP condition, the model may also be valuable to studies aimed at elucidating disease mechanisms.

## Figures and Tables

**Table 1:** Disease scoring.

Phenotype	Readout	Outcome	Score
Anorexia	Weight	No change or increase in weight	0
		1–5% decrease in weight	1
		6–10% decrease in weight	2
		11–15% decrease in weight	3
		>15% decrease in weight	4
	Food intake	10–15 g of diet eaten	0
		3–9 g of diet eaten	1
		0–2 g of diet eaten	2

Malaise	Nest building	Nestlet shredded 80–100%	0
		Nestlet shredded 20–79%	1
		Nestlet shredded 0–19%	2
	Activity	Mouse runs away when hand is placed behind it to pick it up by the tail	0
		Mouse moves away when hand is placed behind it to pick it up by the tail	1
		Mouse is reluctant to move even when touched	2
		Mouse is found dead	3

Pain	Posture	Normal gait	0
		Modestly curved back when walking	1
		Back curved and moves with front and hind legs closely together	2
	Nose bulge	Head is pointy and hair from the nose to the ears is lying flat	0
		Hair from the nose to the ears is slightly raised	1
		Hair from the nose to the ears is standing up giving the appearance of a different head shape	2
	Orbital tightening	Eyes are open and nearly circular	0
		Slight squint	1
		Eyes nearly closed	2

Skin inflammation	Appearance	Skin looks normal	0
		Skin appears dry	1
		Skin is red and flaky	2

## Abbreviations

GPP: Generalized pustular psoriasis
IL: Interleukin
Ra: Receptor antagonist.

## References

[1] Marrakchi S., Guigue P., Renshaw B. R. (2011). Interleukin-36-receptor antagonist deficiency and generalized pustular psoriasis. *The New England Journal of Medicine*.

[2] Onoufriadis A., Simpson M. A., Pink A. E. (2011). Mutations in IL36RN/IL1F5 are associated with the severe episodic inflammatory skin disease known as generalized pustular psoriasis. *American Journal of Human Genetics*.

[3] Tauber M., Bal E., Pei X.-Y. (2016). IL36RN mutations affect protein expression and function: a basis for genotype-phenotype correlation in pustular diseases. *Journal of Investigative Dermatology*.

[4] Jensen L. E. (2010). Targeting the IL-1 family members in skin inflammation. *Current Opinion in Investigational Drugs*.

[5] Towne J. E., Garka K. E., Renshaw B. R., Virca G. D., Sims J. E. (2004). Interleukin (IL)-1F6, IL-1F8, and IL-1F9 Signal Through IL-1Rrp2 and IL-1RAcP to activate the pathway leading to NF-*κ*B and MAPKs. *Journal of Biological Chemistry*.

[6] Milora K. A., Fu H., Dubaz O., Jensen L. E. (2015). Unprocessed interleukin-36*α* regulates psoriasis-like skin inflammation in cooperation with interleukin-1. *Journal of Investigative Dermatology*.

[7] Uribe-Herranz M., Lian L.-H., Hooper K. M., Milora K. A., Jensen L. E. (2013). IL-1R1 signaling facilitates Munro's microabscess formation in psoriasiform imiquimod-induced skin inflammation. *Journal of Investigative Dermatology*.

[8] Foster A. M., Baliwag J., Chen C. S. (2014). IL-36 promotes myeloid cell infiltration, activation, and inflammatory activity in skin. *The Journal of Immunology*.

[9] Tominaga C., Yamamoto M., Imai Y., Yamanishi K. (2015). A case of old age-onset generalized pustular psoriasis with a deficiency of IL-36RN (DITRA) treated by granulocyte and monocyte apheresis. *Case Reports in Dermatology*.

[10] Sugiura K., Haruna K., Suga Y., Akiyama M. (2014). Generalized pustular psoriasis caused by deficiency of interleukin-36 receptor antagonist successfully treated with granulocyte and monocyte adsorption apheresis. *Journal of the European Academy of Dermatology and Venereology*.

[11] Rossi-Semerano L., Piram M., Chiaverini C., De Ricaud D., Smahi A., Koné-Paut I. (2013). First clinical description of an infant with interleukin-36-receptor antagonist deficiency successfully treated with anakinra. *Pediatrics*.

[12] Viguier M., Guigue P., Pagès C., Smahi A., Bachelez H. (2010). Successful treatment of generalized pustular psoriasis with the interleukin-1-receptor antagonist anakinra: lack of correlation with IL1RN mutations. *Annals of Internal Medicine*.

[13] Hüffmeier U., Wätzold M., Mohr J., Schön M. P., Mössner R. (2014). Successful therapy with anakinra in a patient with generalized pustular psoriasis carrying IL36RN mutations. *British Journal of Dermatology*.

[14] Skendros P., Papagoras C., Lefaki I. (2016). Successful response in a case of severe pustular psoriasis after interleukin-1*β* inhibition. *British Journal of Dermatology*.

[15] Flutter B., Nestle F. O. (2013). TLRs to cytokines: mechanistic insights from the imiquimod mouse model of psoriasis. *European Journal of Immunology*.

[16] Langford D. J., Bailey A. L., Chanda M. L. (2010). Coding of facial expressions of pain in the laboratory mouse. *Nature Methods*.

[17] Kushner I., Rzewnicki D. L. (1994). The acute phase response: general aspects. *Bailliere's Clinical Rheumatology*.

[18] Whitacre C. C., Reingold S. C., O'Looney P. A. (1999). A gender gap in autoimmunity. *Science*.

[19] Dinarello C. A. (2004). Review: infection, fever, and exogenous and endogenous pyrogens: some concepts have changed. *Journal of Endotoxin Research*.

[20] Hori T., Oka T., Hosoi M., Abe M., Oka K. (2000). Hypothalamic mechanisms of pain modulatory actions of cytokines and prostaglandin E2. *Annals of the New York Academy of Sciences*.

[21] Walter A., Schäfer M., Cecconi V. (2013). Aldara activates TLR7-independent immune defence. *Nature Communications*.

[22] Stelzner K., Herbert D., Popkova Y. (2016). Free fatty acids sensitize dendritic cells to amplify TH1/TH17-immune responses. *European Journal of Immunology*.

[23] Tortola L., Rosenwald E., Abel B. (2012). Psoriasiform dermatitis is driven by IL-36-mediated DC-keratinocyte crosstalk. *The Journal of Clinical Investigation*.

[24] van der Fits L., Mourits S., Voerman J. S. A. (2009). Imiquimod-induced psoriasis-like skin inflammation in mice is mediated via the IL-23/IL-17 axis. *The Journal of Immunology*.

[25] McColl A., Thomson C. A., Nerurkar L., Graham G. J., Cavanagh J. (2016). TLR7-mediated skin inflammation remotely triggers chemokine expression and leukocyte accumulation in the brain. *Journal of Neuroinflammation*.

[26] Wang P., Meinhardt B., Andre R. (2005). The interleukin-1-related cytokine IL-1F8 is expressed in glial cells, but fails to induce IL-1*β* signalling responses. *Cytokine*.

[27] Berglöf E., Andre R., Renshaw B. R. (2003). IL-1Rrp2 expression and IL-1F9 (IL-1H1) actions in brain cells. *Journal of Neuroimmunology*.

[28] Bozoyan L., Dumas A., Patenaude A., Vallières L. (2015). Interleukin-36*γ* is expressed by neutrophils and can activate microglia, but has no role in experimental autoimmune encephalomyelitis. *Journal of Neuroinflammation*.

[29] Lovenberg T. W., Crowe P. D., Liu C. (1996). Cloning of a cDNA encoding a novel interleukin-1 receptor related protein (IL1R-rp2). *Journal of Neuroimmunology*.

[30] Towne J. E., Renshaw B. R., Douangpanya J. (2011). Interleukin-36 (IL-36) ligands require processing for full agonist (IL-36*α*, IL-36*β*, and IL-36*γ*) or antagonist (IL-36Ra) activity. *The Journal of Biological Chemistry*.

